# Effectiveness of the first French psychoeducational program on unipolar depression: study protocol for a randomized controlled trial

**DOI:** 10.1186/s12888-015-0667-7

**Published:** 2015-11-17

**Authors:** Déborah Ducasse, Philippe Courtet, Maude Sénèque, Catherine Genty, Marie-Christine Picot, Raymund Schwan, Emilie Olié

**Affiliations:** Department of Emergency Psychiatry and Post Acute Care, CHU Montpellier, Hôpital Lapeyronie, 371 Avenue Gaston Giraud, 34295 Montpellier, France; Inserm, U1061 Montpellier, University of Montpellier, Montpellier, France; FondaMental Foundation, ᅟ, France; Clinical & Epidemiological Reseach Unit, CHU, Montpellier, France; Psychotherapeutic center of Nancy, CHU, Nancy, France

**Keywords:** Psychoeducational program, Depression, Treatment adherence, Remission

## Abstract

**Background:**

Major Depressive Disorder (MDD) is highly prevalent and was associated with greater morbidity, mortality (including suicide), and healthcare costs. By 2030, MDD will become the leading cause of disability in high-income countries. Notably, among patients with a previous experience of a major depressive episode, it was indeed estimated that up to 85 % of those patients will suffer from relapse. Two main factors were associated with a significantly higher risk of relapse: poor medication adherence and low self-efficacy in disease management. Interestingly, these issues could become the targets of psychoeducational programs for chronic diseases. Indded psychoeducational program for depression are recommended in international guidelines, but have not yet been proposed in France.

**Methods/Design:**

We propose to evaluate the first French psychoeducational program for depression “ENVIE” in a multicenter randomized controlled trial. The group intervention will include 9 weekly sessions. Its aim is to educate patients on the latest knowledge on depression and effective treatments through didactic and interactive sessions. Patients will experiment the latest innovating psychological skills (from acceptance and commitment therapy) to cope with depressive symptoms and maintain motivation in behavioral activation. In total, 332 unipolar non-chronic (<2 years) outpatients with moderate to severe depression, without psychotic features, will be randomly allocated to the add-on ENVIE program (*N* = 166) or to a waiting list (*N* = 166). The follow-up will last 15 months and include 5 assessment visits.

The primary endpoint will be the remission rate of the index episode at 15 months post-inclusion, defined by a Montgomery and Asberg Depression Rating Scale (MADRS) score ≤ 12 over an 8-week period, and without relapse during follow-up. We will also assess the response rate and relapse at 15 months post-inclusion, hospitalization rate and adherence to treatment during the follow-up period, quality of life and global functioning upon inclusion and at 9 and 15 months post inclusion.

**Discussion:**

If the proposed trial shows the effectiveness of the intervention, but also an increased remission rate in depressed outpatients at 15-months post-inclusion, in addition to improved treatment adherence in patients, it will further promotes arguments in favor of a wide dissemination of psychoeducational programs for depression.

**Trial registration:**

This trial is registered under number 2015-A00249-40 (PURE clinical trial: NCT02501226) (June 30th, 2015).

## Background

MDD is associated with a considerable burden on individuals and societies and a lifetime prevalence of 12 % in men and 26 % in women [1], MDD ranks as the fourth disorder having the highest disease burden, accounting for 40.5 % of DALYs caused by mental disorders [2]. MDD will be the leading cause of disability in high-income countries by 2030. MDD has been associated with greater morbidity, mortality (including suicide), healthcare utilization and costs [3]. Economic costs of depression have doubled in the past ten years, mainly due to an increase in indirect costs from loss of productivity [4, 5]. There is a clear need to improve MDD management without increasing public mental health costs.

Although clinical improvement is often achieved in 60–70 % of depressed patients treated with antidepressants, only about 35 % will reach remission [6, 7]. In addition to antidepressants, complementary therapeutic strategies are needed. Furthermore, among patients who experienced one major depressive episode, it is estimated that up to 85 % of them will relapse [1]. Moreover, patients who experienced two episodes, have a 70–90 % risk of having a third episode [1] The probability of subsequent episodes increases with the number of previous episodes, with a shorter interval between recurrences [8]. These data highlight the need to improve preventive actions to limit depression recurrence. Two main factors have been associated with a significantly higher risk of recurrence: poor medication adherence and low self-efficacy to manage one’s illness (i.e. the patient's confidence in his or her ability to engage in behaviors to manage and prevent further recurrences) [1]. Interestingly, these two factors are targets of psychoeducational programs for chronic diseases. Indeed, these types of programs are designed to educate patients on topics that are essential and can empower them to become active in their recovery process through knowledge and information. Benefits of empowerment include improved self-image, self-efficacy, as well as increased ability to cope with daily life and a higher likelihood of reaching treatment goals [9].

Even if most research on psychoeducation for patients with mood disorders was conducted in bipolar disorder [10], international clinical practice guidelines recently recommended psychoeducational interventions in MDD [11–13]*.* Psychoeducation in MDD was shown to: 1) reduce depressive symptoms [5, 14–29], 2) reduce the risk of relapse or recurrence [26, 30–33], 3) improve treatment adherence [17, 18, 34], 4) increase quality of life [29] and global functionning [25, 35]. In these studies, the program “Coping with depression” (CWD) was the most used. CWD is a cognitive behavioral psychoeducative intervention based on the theory of depression and social learning [36], which aims at improving self-esteem, social skills, and management of depressive thoughts (skills in restructuring negative cognition). A recent meta-analysis [26] that included 25 randomized controlled trials showed that CWD was effective in reducing depressive symptoms (overall mean effect size of 0.28 (95 % CI:0.18–0.38)), and in preventing relapses (overall mean effect size of 0.62 (95 % CI: 0.43–0.91)). But CWD is complex and requires up to 16 sessions. Thus, other psychoeducational formats have been developed: psychoeducational cognitive workshops [14, 15, 19] and videotaped educational materials [37, 38]. However, most studies had a short-term follow-up limiting the interpretation of the effectiveness of these psychoeducational programs on the long-term management of MDD.

To our knowledge, no psychoeducational program for MDD is available in the French language. Thus, even though international guidelines recommend integrating a psychoeducational program in MDD treatment, none has yet been made available in France. We have developed the first French psychoeducational program for depression called “ENVIE”. This group intervention consists of 9 weekly sessions. It aims at teaching patients the latest knowledge on depression and effective treatments, through didactic and interactive sessions. Patients will experiment the last innovating psychological skills to cope with depressive symptoms and maintain their motivation in behavioral activation [39, 40]. Moreover, the latest innovating psychological skills, which will be taught in the “ENVIE” program, belong to Acceptance and Commitment Therapy (ACT) [41]. Interestingly, ACT has shown equal to superior effectiveness compared to traditional cognitive behavioral therapy on unipolar depression [42–45]. Additionally, the specific impact of ACT skills was recently reported on suicidal risk [46, 47], which was highly associated with depression. Finally, patients will become experts and actors in the management of their disease in order to enhance treatment adherence, since lack of the latter has been strongly associated with an increased risk or relapse.

### Objective and research questions

Primary objective:

The main objective is to compare the effectiveness (rate of remission of index episode at 15 months post inclusion, without relapse during follow-up) of the add-on ENVIE psychoeducational program vs. regular treatment only, in unipolar non-chronic (<2 years) outpatients with moderate to severe depression, without psychotic features.

Secondary objectives:

Comparing add-on psychoeducational program with treatment as usual in regards to:

- Decrease in depression intensity between inclusion and at 3, 6, 9 and 15 months post inclusion;
- Response rate 15 months after inclusion;
- Relapse rate 15 months after inclusion;
- Hospitalization rate during the follow-up period;
- Treatment adherence (antidepressants) between inclusion and at 3, 6, 9 and 15 months after inclusion;
- Changes in quality of life and global functioning between inclusion and at 9 and 15 months after inclusion;
- Modification of psychotropic treatments between inclusion and at 15 months post inclusion.

## Methods

### Design

We will conduct a two-arm randomized controlled trial, in order to compare the effectiveness of add-on psychoeducational program for unipolar depression vs. treatment as usual in depressed outpatients. This trial will take place in 11 French centers.

Based on medical records, eligible unipolar outpatients suffering from non-chronic (duration of current episode <2 years) major depressive episode (DSM-IV criteria) of moderate to severe intensity (MADRS score >24) will first be screened in each center. During the inclusion visit, participants matching all inclusion criteria and presenting no exclusion criteria will be informed by the investigator and invited to sign a consent form. They will be randomized in one of the two arms: add-on psychoeducational program (interventional group) or waiting list (control group). An independent researcher, not otherwise involved in the study, will perform the allocation. Each patient will be followed during 15 months with 5 visits: at baseline (V1), 3 months (V2), 6 months (V3), 9 months (V4) and 15 months (V5) after baseline. Blinded and trained evaluators will assess patients during the follow-up.

This research, involving human subjects, has been performed in accordance with the Declaration of Helsinki. The study protocol has been approved by an appropriate ethics committee (CPP Sud Méditerranée IV) (covering all 11 participating centres) and is registered under number 2015-A00249-40 (PURE clinical trial: NCT02501226) (June 30th, 2015).

### Sample size

For the control group the full remission rate at 15 months is expected to be 30 % [48]. If, in the experimental group, this rate is 15 % higher, with a power of 0.80 and a two-tailed significance level at 0.05, the sample size ends up to 166 patients per group. Because loss to follow-up will be considered as “not in remission”, no adjusted sample size for taking dropouts into account has to be established. Thus, a total number of 332 subjects will be enrolled in the study. Each center will recruit between 20 and 40 patients: one to 2 psychoeducational groups of 8–10 patients and 10–20 patients in the control group.

### Participants and procedure

#### Inclusion and exclusion criteria

We will include outpatients [1] aged 18–65 years [2] with a main diagnosis of non-psychotic non-chronic (<2 years) major depressive episode (DSM-IV criteria) of moderate to severe intensity (Montgomery Asberg Depression Scale score >24) [49] taking at least one antidepressant [4] able to speak, read and understand French [5] and able to give written informed consent.

We will exclude subjects [1] with a current diagnosis of substance abuse or dependence in the 6 months prior to inclusion, excluding tobacco, [2] current psychotic features, [49] duration of current depressive episode >2 years, [4] current organic mental disorder or mental retardation, or severe comorbid medical condition, [5] lifetime history of schizophrenia, or schizoaffective or bipolar disorder, manic, hypomanic, or mixed episodes according to DSM-IV criteria, [6] sensory or cognitive disabilities, [7] hospitalized full-time at the time of inclusion, [8] having a relationship or being employed by the sponsor or investigator. We will also exclude patients who are planning a long stay outside the region preventing compliance with the scheduled visits, and subjects participating in another trial.

#### Recruitment

Participants will be recruited via medical consultations in the 11 French investigation centers.

Written informed consent will be obtained from participants.

#### Eligibility assessment and randomization

People who apply for participation in the study will receive an information letter with comprehensive details on study procedures. Signed written informed consent will be obtained from all participants. They will be informed that they can withdraw from the intervention and/or study at any time without any negative consequences.

The first visit will consist in evaluating the following data:

- ○ Sociodemographic characteristics: age, gender, nationality, marital status, educational level, employment status;
- ○ Psychopathology using the Mini-International Neuropsychiatric Interview (M.I.N.I.) [50];
- ○ Number of previous depressive episodes, date of onset of the current episode;
- ○ Depression intensity using the Montgomery Asberg Depression Scale (MADRS) and Beck Depression Inventory (BDI);
- ○ Global functioning using the Functioning Assessment Short Test (FAST);
- ○ Quality of life using the World Health Organization Quality Of Life measure-26 (WHOQOL-26) scale;
- ○ History of psychotropic treatment (name, dose);
- ○ Treatment adherence using the Medication Adherence Rating Scale (MARS);
- ○ Lifetime suicidality using the Columbia–Suicide Severity Rating Scale (C-SSRS).

All inclusion and non-inclusion criteria will be checked by the study investigator. Patients will then be informed of their eligibility and randomized with the Capture Software System (Clinsight). Randomization will be performed with a permuted-block 1:1 ratio randomization list with varying block sizes and will be stratified in participating centers. Allocation concealment will be achieved by a centralized randomization procedure via an electronic case-report form.

#### Assessments

Patients will be assessed by clinicians blinded to treatment allocation, at inclusion (1–3 weeks before the intervention: visit 1) and at 3 months (end of the psychoeducational program: visit 2), 6 months (visit 3), 9 months (visit 4) and 15 months (visit 5) after inclusion. Patients will be advised to refrain from telling which group they were allocated to during visits.

**Fig. 1:**
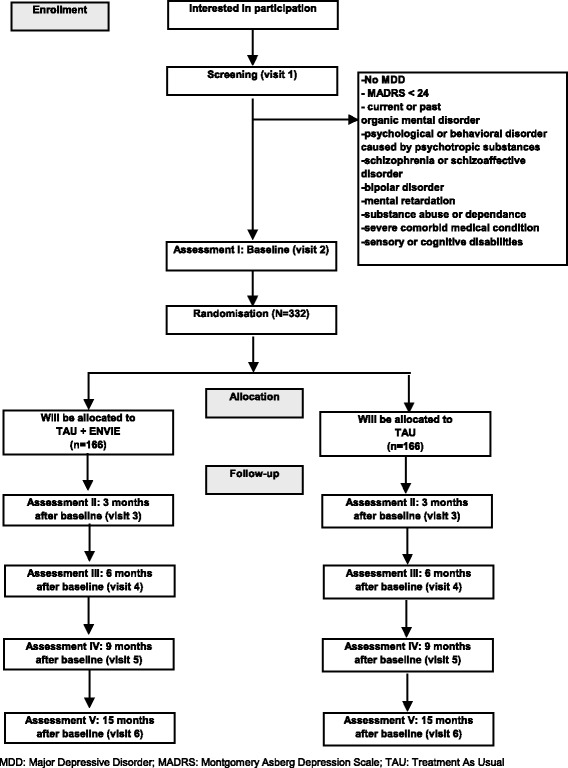
Study flow

See Fig. 1 for a detailed overview.

Investigators should make every effort to minimize the number of patients lost to follow-up and obtain a maximum of information on patients lost to follow-up, particularly in looking for any adverse events.

To limit patients lost to the touch, participants in the intervention group will be sent a text message on their mobile phone (internet-based free site) one day before each psychoeducational session. Each participant will receive two text messages: one the week before and the other the day prior to each follow-up visit.

## Intervention

### ENVIE: the first French psychoeducational group program

#### Add-on psychoeducational group program

The intervention will consist of 9 weekly, 90-min sessions led by two trained educators (nurse and medical doctor). Each group will consist of 8–10 participants. The ENVIE program will provide participants with the latest medical knowledge on depression and effective treatments in a didactic manner [51–57], as well as the latest innovating psychological skills to cope with depressive symptoms and maintain motivation in behavioral activation [39, 40, 58]. These psychological skills belong to Acceptance and Commitment Therapy [58].

The program includes 9 sessions, each focusing on a specific theme or skill:

1. Presentation of the program and the matrix, and psychotherapeutic tools used during the entire program to motivate patients to behave in a manner that is consistent with what is important in their life [58].
2. Functional analysis of depression using the matrix (patients are taught to understand which symptoms/behaviors are linked to depression, why they feel they are struggling, and what they can do to stop the struggle)
3. Causes leading to depression
4. Consequences of depression and comorbidities
5. Neurobiological bases of depression
6. Self-analysis of mood state, recognition of prodromal symptoms of relapse
7. Information about antidepressants, and evidence-based medical treatments for unipolar depression
8. Lifestyle recommendations
9. Behavioral activation skills

During sessions, participants will be encouraged to raise questions about anything they want to know about depression. To enhance the active role of patients, each session will be followed with take-home homework.

One or two psychoeducational groups of 8 to 10 patients will be planned in each center.

#### Treatment as usual

Treatment as usual will consist of the usual clinical management of depression including psychiatric evaluation and adaptation of pharmacological treatment. There will be no treatment restriction during the study period. The current pharmacological treatment will be recorded at each visit.

## Endpoints

### Safety endpoints

Incidence, relatedness, and severity of treatment-emergent adverse events will be evaluated at each visit until the end of study. Safety parameters assessed in this clinical trial will be recorded and evaluated through repeated clinical examinations of the patients. It relies on assessment of suicidality using the Columbia–Suicide Severity Rating Scale (C-SSRS) [59]: rate of suicidal behaviors (completed suicide and suicide attempts) at 15 months post inclusion, variation of severity and intensity of suicidal ideations subscores between baseline and at 3, 6, 9 and 15 months after inclusion. If a patient exhibits a high-risk of suicide (increased suicide ideations, planning a suicide attempt) during a visit, investigators will contact the psychiatrist in charge of this patient.

Moreover, acceptability will be assessed. It will consist in the number of missed sessions, and satisfaction at 3 months post inclusion using a Likert scale ranging from 0 (not useful at all) to 10 (extremely useful) for the intervention group.

### Efficacy endpoints

#### Primary endpoint

Remission rate of the index episode at 15 months after inclusion, defined by a Montgomery and Asberg Depression Rating Scale (MADRS) [60] score ≤ 12 for over 8-weeks, without relapse during the follow-up period.

#### Secondary endpoints

- Variation of the depression intensity between baseline, and at 3, 6, 9 and 15 months after inclusion using MADRS and Beck Depression Inventory (BDI) [60, 61];
- Evolution of MADRS and BDI scores during follow-up;
- Response rate (50 % decrease in MADRS score) at 15 months after inclusion;
- Relapse rate (MADRS >12 after remission of the index episode) at 15 months after inclusion;
- Hospitalization rate during follow-up;
- Variation of global functioning, using the Functioning Assessment Short Test (FAST) [62], between baseline, and at 9 and 15 months after inclusion;
- Variation of quality of life using the World Health Organization Quality Of Life measure-26 (WHOQOL-26) scale [63] between baseline, and at 9 and 15 months after inclusion;
- Variation of treatment adherence using the Medication Adherence Rating Scale (MARS) [64] between baseline and 15 months after inclusion;
- Variation of benzodiazepines doses between baseline and at15 months after inclusion;
- Discontinuation rate of the antidepressant treatment at 15 months after inclusion.

### Statistical analysis

#### General statistical considerations

All statistical analyses will be performed using SAS® (SAS Institute, Cary, NC, USA).

For efficacy variables, we will use a Full Analysis Set, which will include all randomized subjects with a valid primary efficacy baseline measurement and at least one valid primary efficacy measurement. A robustness evaluation using the Completer Set (CS) will also be performed for the primary efficacy variable. The CS is a subset of the FAS and will include all subjects with a valid measurement at the end of the study Period (15 months) for the primary efficacy variable.

A summary of demographic data and baseline characteristics, as well as medical and procedure history will be presented for the whole population (all treatment groups combined) and for each treatment group. For continuous variables, summary statistics (n [number of available measurements], arithmetic mean, standard deviation [SD], median, interquartiles, minimum, and maximum) will be tabulated. For descriptive statistics of continuous variables, change from baseline and each time point will be displayed.

Frequency tables (frequency counts and percentages) will be presented for categorical data.

#### Primary outcome

The outcome of interest is the probability of remission achievement (rate of remission) of the index episode at 15 months post inclusion. Time to remission is defined as the number of days between the date of inclusion and remission or the last follow-up visit in case of no remission (maximum duration: 15 months). But the exact time of this event will not be known (the event could occur during the interval of two visits), so we will use methods for the analysis of interval-censored data. For calculation, all patient lost to follow-up will be considered as not in remission. A Cox proportional hazard model will be used to assess the impact of the intervention (Effect size). Covariates could be added in the Cox’s proportional hazards regression model if both groups cannot be compared on baseline characteristics alone. The adjusted hazard ratios and their 95 % confidence interval will be reported.

#### Secondary outcomes

The rate of response (decrease of MADRS score by 50 %), and rate of relapse (MADRS >12 after remission of the index episode) at 15 months after inclusion will be analyzed with the similar method used for the main criterion.

A mixed-model will be used to analyze:

1. Variations (intra- and between-group) of depression intensity using MADRS and BDI scores between inclusion, and at 3, 6, 9 and 15 months after inclusion.
2. And model the evolution of MADRS and BDI scores during follow-up
3. Variations (intra- and between-group) of global functioning (using the FAST) between inclusion, and at 9 and 15 months after inclusion,
4. Variations of quality of life using WHOQOL-26 scale between inclusion, and at 9 and 15 months after inclusion,

The rate of relapse (MADRS >12 after remission of the index episode) at 15 months after inclusion, the rate of hospitalization during follow-up and the discontinuation rate of antidepressant therapy at 15 months after inclusion will be analyzed using a chi-square test or Fisher’s exact test if the chi-square is not valid.

The between-group comparison on variation in treatment adherence using MARS and changes in benzodiazepines doses between inclusion and 15 months post inclusion will be performed using the student-t test if distribution is normal or the Mann–Whitney test, if otherwise.

#### Safety analyses

For the analysis of safety data (AEs, vital signs), RS will be used. Adverse events occurring during this study will be presented by system organ class (SOC), high level term (HLT), and preferred term [65] in a frequency table giving the number of events, number of subjects, and percentage of subjects who experienced the event by treatment group.

Subjects with multiple AEs will be counted only once within each PT, HLT, and SOC. Coding of the AEs will be performed with the Medical Dictionary for Regulatory Activities.

Vital signs variables and their changes from Baseline will be presented using descriptive statistics by visit and treatment group.

Rate of suicidal behaviors (completed suicide and suicide attempts) at 15 months after inclusion, variation of severity and intensity of suicidal ideations subscores between inclusion and at 3, 6, 9 and 15 months after inclusion will be reported for each group.

## Discussion

Major Depressive Disorder is a highly prevalent disorder associated with a considerable loss of quality of life, increased mortality rates, and substantial economic costs. However, because of a lack of knowledge about this disease, many patients remain untreated although they need help. Also, among patients treated with adequate medication, only 35 % will reach remission. Furthermore, the probability of MDD increases with the number of previous episodes, and the interval between recurrences does decrease with each new episode. However, two main factors associated with a significantly higher risk of relapse are: poor treatment adherence and low self-efficacy to manage depression, which are the primary targets in psychoeducation. Thus, psychoeducation has shown its effectiveness in reducing depressive symptoms and the risk of relapse or recurrence and in increasing patients’ quality of life and global functioning. Moreover, psychoeducation is a simple add-on treatment, which can be delivered by any healthcare professional thus reducing costs and increasing the number of patients able to benefit from this program. Currently, few programs have been developed and those are mainly available in the English language.

This study will evaluate the effectiveness of the first French psychoeducational program associated with treatment as usual, via the rate of remission on depressed outpatients at 15 months of follow-up. A special emphasis will be given to teaching didactically the latest scientific knowledge about depression and effective treatments, and the latest innovating psychological skills to cope with depressive symptoms and maintain motivation in behavioral activation. Notably, psychological skills stem from Acceptance and Commitment Therapy, which is a brief integrative therapy (third wave behavior therapy, motivational interviewing, and existential therapy) with validated effectiveness on depression and suicidal risk. Finally, the ENVIE program has already been conducted in the primary investigator centre, showing high adherence and satisfaction rates.

This study will have some limitations. First, as in most longitudinal studies we will need to deal with the problem of missing values. Thus, since loss to follow-up will be considered as “not in remission”, positive results from this study will be particularly relevant. In order to limit patients lost to follow-up, participants in the intervention group will be sent a text message on their mobile phone one day before each psychoeducational session, and one week and one day before each follow-up visit. Second, patients will be randomized, but there is no double-blind, randomization since the patient will know if he/she belongs to the intervention or control group. Indeed, the control group will consist of a waiting list, and not a control group therapy. Third, we will not have any control over the treatment as usual in each of the two groups.

There will also be several strengths to this study, including the strong methodology of a randomized controlled design, validated assessments conducted by independent raters blinded to treatment condition, a sample defined by a standard diagnostic measure, an appropriate statistical analysis plan, and considering patients lost to follow-up as not in remission. Furthermore, the long-term follow-up (15 months), the assessment of remission and treatment adherence, and the latest innovative scientific didactic content of this short-duration program are highly relevant in these studies. Given these strengths, the results of the study should further enhance the evidence-based data supporting psychoeducational intervention for MDD. Finally, given the 11 French investigator centers implied in this study, the results could be easily generalized to depressed patients visiting French medical centers.

## Conclusion

To overcome the gap between the need for treatment and evidence-based treatment availability and utilisation, cost-effective low-threshold interventions are strongly needed to be accessible to as many people as possible. Psychoeducation is one of them. In fact, a psychoeducational program seems the most available strategy to improve treatment adherence and self-efficacy to manage depression, two main factors associated with the risk of relapse. If the proposed trial shows not only the effectiveness of enhancing the rate of remission in depressed outpatients at 15 months follow-up but also improved treatment adherence in these patients, it would further strengthen the arguments for a wide dissemination of psychoeducational programs for depression.

## Availability of data and materials

All data sets will be presented in additional supporting files.
